# Cacao Pod Husk Flour as an Ingredient for Reformulating Frankfurters: Effects on Quality Properties

**DOI:** 10.3390/foods10061243

**Published:** 2021-05-30

**Authors:** Johannes Delgado-Ospina, Maria Martuscelli, Carlos David Grande-Tovar, Raquel Lucas-González, Junior Bernardo Molina-Hernandez, Manuel Viuda-Martos, Juana Fernández-López, José Ángel Pérez-Álvarez, Clemencia Chaves-López

**Affiliations:** 1 Faculty of Bioscience and Technology for Food, Agriculture and Environment, University of Teramo, Via R. Balzarini 1, 64100 Teramo, Italy; mmartuscelli@unite.it (M.M.); juniorbernardo.molinahernandez@studenti.unite.it (J.B.M.-H.); cchaveslopez@unite.it (C.C.-L.); 2 Grupo de Investigación Biotecnología, Facultad de Ingeniería, Universidad de San Buenaventura Cali, Carrera 122 # 6-65, Cali 76001, Colombia; 3 Grupo de Investigación de Fotoquímica y Fotobiología, Universidad del Atlántico, Carrera 30 # 8-49, Puerto Colombia 081008, Colombia; carlosgrande@mail.uniatlantico.edu.co; 4 IPOA Research Group, Centro de Investigación e Innovación Agroalimentaria y Agroambiental de la UMH (CIAGRO), Miguel Hernández University, Orihuela, CYTED-Healthy Meat. 119RT0568 “Productos Cárnicos más Saludables”, 03312 Alicante, Spain; raquel.lucas@graduado.umh.es (R.L.-G.); mviuda@umh.es (M.V.-M.); j.fernandez@umh.es (J.F.-L.); 5 Faculty of Science, King Abdelaziz University, Jedda 21589, Saudi Arabia

**Keywords:** by-product, co-product, dietary fibers, functional foods, meat products, cacao pod husk flour

## Abstract

The cocoa pod husk is considered a source of dietary fiber with a high content of water-soluble pectins, bioactive compounds which should be viewed as a by-product with the potential to be incorporated into food. This study aimed to investigate the effect of adding different cocoa pod husk flour (CPHF) levels as a starch replacement for reformulating frankfurters. Results showed that the addition of 1.5 and 3.0% pod husk proportionally increased the frankfurter’s fiber content by 0.49 ± 0.08 and 0.96 ± 0.19 g/100 g, which is acceptable for a product that does not contain fiber. Textural properties and sensory characteristics were affected when substituting the starch with CPHF, either totally or partially, although these samples had higher water content, hardness, and adhesiveness while springiness decreased. Non-adverse effects of nitrite on polyphenolic compounds content were evidenced in samples enriched with CPHF. The incorporation of CPHF did not significantly affect the color parameters (ΔE < 3). Finally, the panelists indicated a sensation of the unsalted sausage, suggesting that CPHF may have natural mucoadhesion properties. In conclusion, in formulated meat products such as sausages, plant co-products such as cacao pod husks could be a valid new ingredient to improve technological parameters, functional characteristics, and stability.

## 1. Introduction

The COVID-19 pandemic highlighted the need for healthier dietary patterns, encourage food producers to develop new products. The observatories that exist, globally and nationally, are alert to these changes to help companies re-direct their activities. That is why the pandemic will be an excellent opportunity to develop new products in the world of food, using research and development plus innovation strategies. These strategies are aimed at improving the health and wellness of consumers through products enriched with proteins of high biological value (chia, quinoa, amaranth) [1,2], vitamins, minerals, and, above all, dietary fiber, without forgetting that the environment must be respected [3]. In this context, extracts rich in dietary fiber derived from different parts of plants could be used as would-be functional ingredients due to their potential human health benefits that go far beyond supporting bowel regularity [4]. Moreover, the current consumer increasingly believes that food contributes to their health [5].

The reports of diseases that are directly related to low consumption of fiber in people’s diet, including constipation, diarrhea, ulcerative colitis, diverticulosis, colorectal cancer, cardiovascular disease, and diabetes, are increasing alarmingly [6]. The low fiber consumption depends on eating habits in which foods with low fiber content or easily prepared processed foods to which the fiber has been removed as part of the process are favored. Increasing the incorporation of fiber in food also means looking for new sources of fiber to add to those products already in the market, which is why co-products are an excellent alternative in the search for this type of fiber [7].

Meat is a food group that is not usually a significant source of dietary fiber in the diet as vegetables. Meat products such as comminuted meat products (sausages, mortadella) and hams are no exception, although their formulation incorporates spices that provide small amounts of fiber. However, in ground meat products, there is the possibility of improving their formulation appropriately, managing to incorporate different attributes that allow them to become healthier foods. Several studies are related to meat product innovations. In those studies, several ingredients from spices, fruits, and vegetable by-products were added as bioactive compounds, improving the nutritional, physicochemical, textural, and sensory properties [8,9]. They have even been used as biopreservative additives [10].

Some studies have been carried out showing that some dietary fibers can be successfully incorporated into meat products, making an essential contribution of fiber in the diet and improving some food properties [11,12]. One of the benefits of fiber is that it increases satiety, so when incorporated into processed meat products, it could generate a decrease in the size of the portions, causing the net consumption of meat to decrease and, therefore, the greenhouse gas emissions from livestock. However, the incorporation of fiber is not easy. The incorporation of the fiber depends on the technological properties (concentration, particle size, water-holding capacity, and swelling capacity, among others), physical properties (color it contributes, texture), physicochemical properties (pH, oxidation–reduction potential, activity antioxidant, and prooxidant), chemical properties (composition, type of fiber, etc.), and sensory properties. Even industrial-scale production capacity must be assessed. Therefore, its addition requires extensive scientific and technological studies.

Some by-products, such as cacao pod husk, are important sources of dietary fiber that can be used in processed foods [13]. According to the International Cocoa Organization (ICCO) [14], in the 2019–2020 period, 4,697,000 tons of cocoa were produced globally, representing only 15% of the fruit. The remainder, more than 22,000,000 tons, is represented by pod husk (70 to 75% of the fruit).

Cocoa pod husk (CPH) is considered as a source of bioactive compounds [3,15], antioxidant dietary fiber with a high content (up to 6%) of minerals (K, P, Ca, and Mg) [16], high content of water-soluble pectins [17], and a source of hydrolase enzymes [18]. Its techno-functional properties were recently evaluated, and the high water-holding capacity suggests that it can be used as a good substitute for replacement of emulsifiers or water retainers, incorporating the fiber and antioxidant properties into new food products at the same time [3]. On the other hand, the use of by-products such as cacao pod husk flour generates benefits for the environment. Cacao pod husk is usually left to degrade on the cocoa plantation, causing inoculum of fungal diseases in cacao crops such as black pod rot [3]. In addition, it has resulted in silting, blockage of water drainage systems, and flooding of rivers and other waterbodies.

The ingestion of soluble pectins present multiple benefits. They can increase satiety and decrease caloric intake and adiposity during a high-fat diet, such as when there is consumption of sausages. The objective of the present work was to study the effect of CPH as a partial or total substitute for starch in the reformulation of frankfurters.

## 2. Materials and Methods

### 2.1. Cacao Pod Husk Flour (CPHF)

Fresh cocoa fruits were collected from a Criollo cacao processing farm in Colombia and taken to the laboratory where they were opened for extracting their grains and mucilage. Cocoa pods husks (CPH) were cut into 1 cm wide strips and immediately placed in boiling water for 10 min to inactivate the enzymes, then cooled and taken to undergo a freeze-drying process in a Liophilizer FreeZone 4.5 L (Labconco Corporation, Kansas, USA). Freeze-dried CPH were ground in a Hammer Mill M20 (IKA, Staufen, Germany), passed through a sieve (Dp < 200 µm) to obtain the flour (hereinafter CPHF), and stored at 4 °C until being used in the analyses. The chemical, physical–chemical, and techno-functional properties of CS were investigated in our previous study [3].

### 2.2. Frankfurters Elaboration Process and Treatments

Frankfurters were prepared following a traditional formulation described in [19]. The ingredients were pork shoulder lean meat (70%) and pork backfat (30%). The following percentages of ingredients refer to meat: 15% water (ice form *w*/*w*), 2.5% sodium chloride (*w*/*w*), 1.5% casein, 500 mg/kg sodium ascorbate, 300 mg/kg sodium tripolyphosphate, 150 mg/kg sodium nitrite, 0.5% (*w*/*w*) liquid smoke, 0.2% (*w*/*w*) white pepper, 0.05% (*w*/*w*) nutmeg, and 0.05% (*w*/*w*) coriander powder. For the treatments, the concentrations of CPHF and potato starch (PS) were varied (Table 1).

The frankfurters were prepared at the IPOA Research Group pilot plant at the Miguel Hernández University, Orihuela Spain. Briefly, pork lean meat and pork backfat previously cooled below 12 °C were ground in a cutter (1094-Homogeneizer, Tekator, Höganäs, Sweden) and mixed according to the treatments with the rest of the ingredients for 2 min. After homogenization, the emulsion formed was filled using an EM-12 piston stuffer (Mainca, Granollers, Barcelona, Spain) in cellulose wraps 20 mm in diameter (Fibran, Girona, Spain). The 20 cm long frankfurters were cooked in a water bath (80 °C) until reaching an internal temperature of 72 °C which was verified using a thermocouple probe (Omega Engineering, Inc., Stamford, CT, USA) placed in the geometric center of a sample. The frankfurters were immediately cooled in a blast chiller for 5 min, peeled, vacuum-packed, and stored at 4 °C until used in the analyses [19].

### 2.3. Proximate Composition

The proximate composition was determined in CPHF and frankfurters according to the following AOAC methods for meat: lipid (AOAC 991.36), protein (AOAC 981.10), moisture (AOAC 925.45), ash (AOAC 923.03), and general method for total dietary fiber in foods (TDF) (AOAC 985.29) [20], and the carbohydrate content was determined by calculating the percent remaining after all the other components have been measured (%carbohydrates = 100 − %moisture − %protein − %lipid − %TDF − %ash).

### 2.4. Physicochemical Analysis

The pH of CPHF was measured with an electrode probe connected to a pH meter (GLP 21, Crison, Barcelona, Spain) diluting the CPHF in distilled water (1:10), and the pH of frankfurters was measured for direct penetration into frankfurter at three different points using an electrode for penetration measurement. The water activity (a_w_) for CPHF and Frankfurters was measured at 25 °C using a water activity analyzer (Novasina TH-500, Pfaeffikon, Zürich, Switzerland).

### 2.5. Residual Nitrite Level

Residual nitrite level was determined according to standards ISO/DIS 2918.26 [21] with modifications. In brief, ten grams of the previously homogenized samples were placed in an Erlenmeyer flask with 100 mL of water and incubated at 100 °C for 15 min with constant stirring. The samples were cooled in an ice bath, then 2.0 mL of Carrez I reagent and 2.0 mL Carrez II reagent (Merck, Darmstadt, Hesse, Germany) were added, it was left standing for 30 min, its volume was made up to 200 mL in a volumetric flask, and it was filtered through Whatman qualitative filter paper. In a test tube, 5.0 mL of the solution and 5.0 mL of the color reagent (alpha-naphthylamine chloride 0.03% *w*/*v* in acetic acid 20% *v*/*v*) were mixed, left to rest for 20 min, and absorbance at 520 nm determined using a spectrophotometer. The calibration curve was prepared from successive dilutions of a sodium nitrite standard, adding the color reagent in the same way and proportion as the samples. The results were compared against a sodium nitrite calibration curve and expressed as µg NaNO_2_/g sample.

### 2.6. Measurement of Lipid Oxidation: Thiobarbituric Acid Index (TBARS)

The extent of lipid oxidation was determined by measuring the TBARS-reacting substances in frankfurters. Values were expressed as mg of malondialdehyde (MDA)/kg of a sample using the procedure described in [22]. In brief, 2.0 g of sample was homogenized with 16 mL of 10% trichloroacetic acid (TCA) for 15 min. The sample was placed at rest for 30 min in an ice bath. The sample homogenized was filtered through Whatman qualitative filter paper (grade 1) into 25 mL Erlenmeyer flasks. Filtered solution (2 mL) was mixed with 2 mL of 0.5% thiobarbituric acid (TBA) in distilled water in capped test tubes. Tubes were incubated in boiling water for 35 min. The absorbance was determined at 532 nm against a blank containing 2 mL of 10% TCA and 2 mL of 0.5% TBA solution. The calibration curve was prepared from successive dilutions of a malonaldehyde standard (prepared by acid hydrolysis of 1,1,3,3-tetramethoxypropane), adding the TBA reagent in the same way and proportion as the samples.

### 2.7. Color, Reflectance Spectra and Reflectance Ratios

Colorimetric analysis was performed using a CM-700d Spectrophotometer (Konica Minolta, Osaka, Japan). The CIELAB color coordinates (L*, a*, and b*), chroma C* (Equation (1)), hue angle *h_ab_* (Equation (2)), color difference ΔE* (Equation (3)), and reflectance spectra (360 nm–740 nm) were determined with the following settings: illuminant D65, observer 10°. Guidelines for meat color evaluation were applied [23] and the method of [24] was followed. For the CPHF, it was deposited in a rectangular cell of 20 mm path length (CM-A132) and the measurement was carried out on both sides of the cell, while the frankfurters were cut into several 2 cm cross sections for the analysis. The reported values correspond to the average of four measurements. The reflectance ratios R630/R580, R650/R570, and R560/R500 were calculated from the reflectance values obtained for the wavelength defined for each.

(1) $$C^{*}=\sqrt{a^{*2}+b^{*2}}$$

(2) $$h_{ab}=\arctan\frac{b^{*}}{a^{*}}$$

(3) $$\Delta E^{*}=\sqrt{{\left(\Delta L^{*}\right)}^{2}+{\left(\Delta a^{*}\right)}^{2}+{\left(\Delta b^{*}\right)}^{2}}$$

### 2.8. Textural Properties

Texture profile analysis was performed out on frankfurters according to the methodology used in [19]. A Texture Analyzer TA-XT2i (Stable Micro Systems, Surrey, England) was used. Frankfurter cross-sections (15 mm long) were subjected to a two-cycle compression to 75% deformation of their original height with an activation force of 5 g, speed of 5 mm/s, and 5 s retrieval between cycles. The following attributes were obtained: hardness (N), adhesiveness, springiness (mm), cohesiveness (Nmm), gumminess, chewiness (Nmm), and resilience. Four determinations were made per sample.

### 2.9. Sensory Evaluation

The frankfurters were cooked, and were tested after storage at 4 °C (Fridge, Siemens BSH, Zaragoza, Spain) for two days to evaluate color, taste (saltiness), texture (compactness, greasiness, juiciness, and hardness), flavor, and general acceptability using 25 sensory assessors untrained in the sensory analysis of frankfurters (12 males and 13 females) aged 18–55 years, according to protocols described in [19]. In brief, four pieces of 2.0 cm width (one from each treatment) were cut from the frankfurters and served with unsalted crackers and mineral water to clean the palate between samples (all served at room temperature). This analysis was performed under white fluorescent lights in individual booths, with the room temperature kept at 25 °C. The hedonic scale consisted of 9 levels (1—dislike extremely and 9—like extremely).

### 2.10. Statistical Analysis

Three independent experiments were made (10 kg of sausages for each experiment were elaborated), three replications of each factor and level were made, and three repeats were analyzed for each sample. The analysis of variance (ANOVA) was performed to determine the statistical significance (*p* < 0.05) of adding different concentrations of CPHF and PS: control (3.0% PS); CPHF 1.5 (1.5% CPHF); CPHF 3.0 (3.0% CPHF); and CPHF-PS 1.5 (1.5% PS and 1.5% CPHF). The Tukey HDS test (*p* ≤ 0.05) was used for means comparisons. Data are presented as mean values ± standard deviation (SD). The Statgraphics Centurion XVI program (Statgraphics Technologies, Virginia, USA) was used for these statistical analyses.

## 3. Results and Discussion

### 3.1. Proximate Composition of Frankfurters

The compositional analysis of CPHF was previously reported in [3] and is presented in Table 2 to understand the frankfurter results. The technological properties depend significantly on the material’s chemical composition, mainly due to the chemical interactions between proteins, lipids, and fiber with water and oil molecules. In CPH, the composition reported by different authors varies widely. However, the protein and lipid values are within the reported ranges in the literature [15]. The soluble fiber content, which corresponds to 30% of the total dietary fiber, is mainly attributed to the presence of pectins [25].

As expected, the addition of CPHF increased the frankfurter’s fiber content (*p* < 0.05). Thus, the contribution from the fiber to the nutritional content of the sausage is essential. Similarly, a significant increase in protein content was found, consistent with the content provided by CPHF. A significant increase was found concerning the control, related to the high water-holding capacity (WHC) of the CPHF (28.90 ± 1.82 g/g) found by us in a previous study [3], which increases the hydration of the sausage. Similar results are found with the addition of chia seed flour to sausages [19].

The lipids showed a significant decrease (*p* < 0.01) concerning the control. This decrease is related to the low oil-holding capacity (OHC) of the CPHF (1.99 ± 0.02 g/g) found by us in a previous study [3], which slightly decreases the ability to stabilize the emulsion, which can be quickly released during cooking. This trend can be something positive from the point of view of having foods with lower fat content as long as it does not affect the other sensory and texture characteristics of the product. Proximate composition data expressed on a dry matter is shown in Table S1.

### 3.2. Physicochemical Analysis. pH and a_w_

The addition of the CPHF to the frankfurters did show significant differences (*p* < 0.01) in the pH concerning the control, although the pH of the CPHF is slightly lower. A decrease in the treatment that combines starch and CPHF (CPHF-PS 1.5), due to starch–protein interactions being favored, leaves the CPHF groups without interaction, which causes the carboxyl groups to be exposed, showing a decrease in the pH of the sausage. All sausages showed a similar a_w_ value (*p* > 0.05), results that are slightly lower than those reported for other sausages (0.984 a_w_) [19]. Water activity, pH, and temperature are the main parameters that directly impact the growth of microorganisms. Decreasing the water activity in the food limits the growth of microorganisms. In the case of meat products, the growth of bacteria generally occurs due to the high a_w_, and it is not recommended in terms of the sensory properties to take the product to an intermediate humidity (a_w_ 0.6–0.85). Therefore, it is recommended to lower the pH of the product and maintain a low temperature during storage.

### 3.3. Residual Nitrite Level

The use of sodium nitrite in pork processing mainly has important effects on the development of color, acts as an antioxidant additive, prevents warmed-over flavor, and ensures microbiological safety; therefore, the level of residual nitrite is vital to prevent *Clostridium botulinum* spore development, which causes botulism, although decreasing its daily consumption is essential to alleviating the potential risk of carcinogenic, teratogenic, and mutagenic nitroso compounds [26].

Some previous studies have reported that the level of added nitrite in processed meat products decreases over time because nitrite is reduced to nitric oxide (NO), which reacts with myoglobin to form nitric oxide myoglobin [27]. The decrease in nitrite content also depends on factors such as pH, initial nitrite concentration, processing and storage temperatures, the meat/water ratio, and the presence of reducers [28].

In CPHF, residual nitrites levels were not detected. The inclusion of CPHF in the sausage does not alter the residual nitrite level, as is usual when vegetable co-products are used. The addition of vegetables by-products or co-products can incorporate nitrites into meat products since nitrates are naturally found in some parts of plants, especially in green leaves [29]. It has been reported that meat products made with by-products derived from quinoa provide some considerable amount of nitrates due to their presence in seeds, which could be used in place of nitrate/nitrite addition in the formulation [1].

The content found in the treatments is higher than 30 mg/kg, which is the lower limit to avoid toxinogenesis under storage conditions, and without exceeding the upper limit of 150 mg/kg [30]. The higher content was found in the control and the CPHF 3.0 treatment, while CPHF 1.5 and CPHF-PS 1.5 presented statistically lower values (Table 2). Although it has been reported that nitrite can react with bioactive compounds, especially with polyphenolic compounds [31], and that CPHF contains large amounts of bioactive compounds [3], no evidence of this was observed in the treatments.

### 3.4. Lipid Oxidation. Thiobarbituric Acid Index (TBARS)

The TBARS values are presented in Table 2. Frankfurters containing CPHF showed a statistically significant increase in TBARS (*p* < 0.05). The sample with the highest co-product content (CPHF 3.0) presented an increase of 105% concerning the control. However, all samples’ TBARS values were lower than the acceptance limit of TBARS for rancidity (0.5 mg MDA/kg) [32]. In this study, the sausages’ TBARS values were slightly higher than those mentioned by Fernández [19], in Frankfurter-type sausages added with chia co-product.

Many factors can influence the degree of oxidation of meat and meat products. However, the rate and extent of oxidation can be retarded, reduced, or prevented by applying natural antioxidants [33]. Although not conclusive, the results obtained contrast with the antioxidant capacity of CPHF (ABTS (139.9 µmol TE g^−1^) and DPPH (132.8 µmol TE g^−1^)) reported in [3], and more in-depth studies should be done in this regard.

In some cases, the antioxidant effect of several types of polyphenol-rich dietary fibers is reversible in animal-based foods. This effect is concentration-dependent; in low concentrations it acts as an antioxidant, and when the concentration increases, the fiber acts as a prooxidant. Moreover, this effect could be the reason that higher CPHF concentrations showed higher TBA values.

### 3.5. Color

The hydrated CPHF showed a significant decrease in the color parameters (L*, a*, C*, *h_ab_*) concerning the dry CPHF. Only the b* value increased. The dry and hydrated CPHF has a pale-yellow color, so the frankfurter color may slightly change when added. Color data are reported in Table 3.

The addition of CPHF to the frankfurters did not show significant changes in L* values, indicating homogeneous incorporation within the meat emulsion in concordance with [34]. On the other hand, the a* and b* values are useful for identifying the evolution of a meat product since they both decrease during oxidation, being the best indicators of metmyoglobin or nitrosyl hemochrome changes during oxidation [35,36]. The CPHF addition did not cause a significant variation in the a* values, while there was an increase in the b* values. In the frankfurter samples, the measurement of the parameters a* and b* suggests that the changes are related to the CPHF color’s contribution and not to oxidative processes. Although the TBA analysis showed slight increases with the addition of CPHF, this oxidative process did not directly affect the color, or the concentration of oxidizing substances was not high enough to affect the color, since it would be “limited” by the antioxidant compounds present in the product.

The increase in CPHF in the frankfurters produced an increase in the C* and *h_ab_* values so that the samples showed a greater intensity of color and the tone changed from red to yellow. This indicates no myoglobin oxidation to metmyoglobin, which occurs when the chroma value decreases and *h_ab_* remains constant [36], reinforcing the argument that the color changes are related to the color contribution of the CPHF.

There were no significant differences in the ΔE* values between the treatments, and these presented values below 2.3. Most reports indicate that values > 3 correspond to changes perceptible by the observer [19]; in this sense, the addition of CPHF produced values below the threshold, which implies that this addition, although not having established values for meat products such as sausages, is not perceptible to the human eye.

In general, the changes presented in the different color parameters are not perceptible, so the CPHF incorporation does not affect frankfurter presentation. Uniformity and color stability are important parameters for the visual acceptance of meat products [23].

#### 3.5.1. Reflectance Spectra

In terms of color, the relationships between dietary fibers, meat matrix, and water are crucial to understanding the color evolution during processing storage and shelf-life. Reflectance spectra are the meat product fingerprint, so it is very important for its evaluation and reflectance ratio.

**Figure 1:**
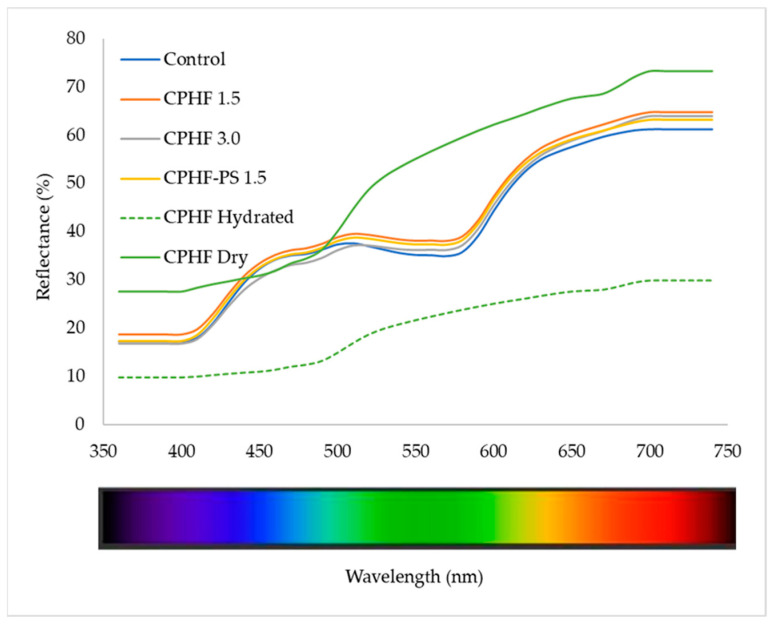
Reflectance spectra (360–740 nm) of cacao pod husk flour (CPHF) (dry and hydrated) and the frankfurters with the addition of CPHF. CPHF Dp < 200 µm.

Figure 1 presents the reflectance spectrum (360–740 nm) obtained from frankfurters and CPHF. A pronounced increase in dry CPHF was observed above 500 nm, indicating a contribution from photosynthetic pigments (green, yellow, and orange pigments) and phenolic compounds. In hydrated CPHF, the spectrum decreased (*p* < 0.05). Hydration can occur by absorption, a process in which water is absorbed into the matrix and the reflection is reduced (diffuse reflectance), which was observed in hydrated CPHF. It can also occur by adsorption, a process in which solid particles with water on the surface reflect more light (specular reflectance) [3].

In the frankfurters, a relative maximum was observed in the blue region (~500 nm), a greater maximum in the red region (~650 nm), and a minimum in the central area corresponding to the green region (~580 nm). The spectrum corresponds to a characteristic spectrum of a cured meat product with which it is possible to calculate the different relationships of wavelengths (ratios), which allow evaluating the changes of the states of the hemopigments during the meat product elaboration process [37].

The oxygenation process also induces a decrease in reflectance in the blue region and increases the red region, changing the meat’s color from purplish red to bright red. In all the treatments, a non-significant increase in reflectance was observed in the entire spectrum compared to the control. Only CPHF 3.0 presented a decrease in reflectance in the blue region (430–500 nm), indicating the beginning of oxygenation of the meat without presenting another distinctive sign of the presence of metmyoglobin.

#### 3.5.2. Reflectance Ratios R630/R580, R650/R570, and R560/R500

Table 4 shows the reflectance ratios, and all treatments present statistically significant differences with respect to the control (*p* < 0.05) but not between CPHF treatments.

The myoglobin and metmyoglobin that have not reacted with nitrite are adequately quantified using the reflectance ratio R630/R580 (pigment transformation index), a ratio of 1.0 would indicate essentially 100% metmyoglobin. During oxidation, this ratio decreases, causing a loss of vibrancy and a brownish appearance [38]. In our reflectance data, this relation for the control is 1.53 ± 0.01 (Table 4). There was a maximum reduction of 0.06 units (CPHF 1.5 and CPHF-PS 1.5), which corresponds to a 1.5% increase in metmyoglobin, and according to Hernandez et al. [36], when 0.2 units of this relationship are reduced, there is a 5% increase in metmyoglobin. A value below the threshold for rejection by consumers (20%).

In the treatments, the values found for the ratio R650/R570 (red color stability index) indicate that the addition of CPHF tends to moderately decrease the cured color, increasing metmyoglobin formation. The red color stability index determines the intensity of the cured color by estimating the relative proportion of reduced (Fe^2+^ pigment) and oxidized (Fe^3+^ pigment) pigments present on the sample’s surface [37]. The values found close to 1.6 indicate a moderate fading towards the metmyoglobin formation.

The reflectance ratio R560/R500 (nitrosation index) determines the relative proportion of native Fe^2+^ pigments and Fe^2+^ pigments stabilized by surface nitrosation, representing the curing process’s efficiency [37]. The statistically higher ratio R560/R500 values found in the treatments concerning the control indicate a lower conversion of myoglobin and oxymyoglobin into nitrosyl myoglobin because of CPHF.

Contrary to what is shown by the color parameters a* and b*, the three indices show that the treatments tend to increase the formation of metmyoglobin (gray) and decrease myoglobin and oxymyoglobin’s conversion into nitrosyl myoglobin (pink) in the frankfurters. In other words, there are minor oxidative processes associated with the incorporation of CPHF, without appreciable changes in color and without jeopardizing the integrity of the product.

### 3.6. TPA Analysis

The TPA analysis performed on frankfurters (Table 5) showed that in most of the parameters, there was significant variation (*p* < 0.05). At this point, the texture parameters impact the initial consumer perception. Hardness increased (81.82 to 94.63 and 86.30 N) when the starch was replaced with the addition of CPHF 1.5% and CPHF 3.0%, respectively (*p* < 0.01) in accordance with [39], who found that the addition of insoluble fiber to sausages increased their hardness, attributed to the ability of some fibers to promote or strengthen connections among the matrix components [40,41]. This textural behavior could be explained by the technological properties of CPHF [3], whose emulsifying ability is probably sufficient to provide stability of the emulsion and gelling properties, helping to maintain the protein network and the continuous phase in the meat emulsion [42]. However, it was noted that when more CPHF is added (3.0%), the hardness decreases. This can be related to its high water-holding capacity (WHC 28.90 ± 1.82 g/g), with more moisture retained in the sausage (Table 2), which causes a decrease in hardness. By replacing only 50% of the starch (CPHF-PS 1.5 treatment), it was noted that the hardness increased to a similar value to the CPHF 3.0 treatment, even with similar water content. This indicates a good integration between fiber and starch within the meat matrix.

Adhesiveness showed a significant increase (*p* < 0.05) with the addition of CPHF, presenting the same tendency for hardness. While springiness presented a trend contrary to hardness, when hardness increased, springiness decreased in the samples. Although the product increases its hardness, it can be easily broken, which can promote chewing. Cohesiveness showed a tendency to decrease with the addition of CPHF (only significant for CPHF 3.0), possibly explained by the greater swelling capacity of CPHF (SWC 14.72 ± 1.13 mL/g), which may decrease the cohesiveness of the sausage. However, when CPHF was mixed with starch, the cohesiveness of CPHF-PS 1.5 increased above the control (not significant). Chewiness and resilience remained constant.

From the point of view of the textural properties, when substituting the starch with CPHF totally or partially, the hardness and the adhesiveness increase are the two most important parameters to consider since they increase significantly.

### 3.7. Sensory Evaluation

**Figure 2:**
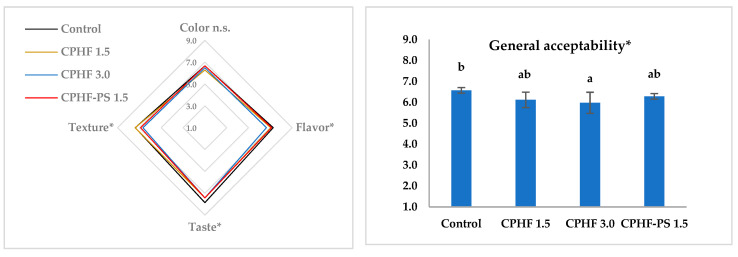
Spider diagram and column diagram of sensory evaluation of frankfurters samples with added cacao pod husk (control, CPHF 1.5%, CPHF 3.0%, and CPHF-PS 1.5%). Mean score of duplicate analysis (*n* = 21). Letters different indicate significant differences (LSD test, *p* < 0.05). Asterisks indicate significance at * *p* < 0.05; n.s. not significant.

In general, the samples were positively evaluated. On a scale of 1 to 9, the scores were between 6.0 and 7.9. It was observed in all aspects that the best-evaluated sample was always the control (Figure 2).

Regarding color, the lowest scores (*p* > 0.05) were for CPHF 1.5 and CPHF 3.0, which are the ones with the most significant color difference, ΔE = 1.82 ± 0.59 and 2.28 ± 0.35 respectively. That is, the panelists were able to detect these color differences. This fact contrasts with the report that in color differences below ΔE = 3, where the observer does not notice the color difference [43], suggesting that the color differences should be evaluated for each particular product and cannot be taken as a standard. In our case, the CPHF-PS 1.5 sample with a ΔE = 1.40 ± 0.08 presented the same score as the control.

Regarding texture, the lowest scores (*p* < 0.05) were in the CPHF 3.0 and CPHF-PS 1.5 samples. The questions about juiciness and hardness were the questions with the lowest score. It is possible that although these samples had higher water content, the water is bound and is not quickly released during chewing and generates a sensation of dryness in the mouth. Additionally, as already mentioned, addition of insoluble fiber to the sausage increases its hardness [39].

For the taste, the panelists gave lower scores (*p* < 0.05) to the samples with CPHF. For the question about the salty taste, the panelists indicated a sensation of unsalted sausage. The addition of CPHF in the sausage can hide the taste of salt, and the polysaccharides in CPHF may have natural mucoadhesion properties. This phenomenon is widely exploited in the pharmaceutical industry to prolong drug residence. It has been investigated to retain taste or aroma molecules in the oral cavity, and other uses [44]. This characteristic is not desirable in food since there is currently a tendency to reduce salt consumption without sacrificing taste [45].

For flavor and general acceptability, the sample with the lowest score was CPHF 3.0, although none of the treatments was rejected and all were judged acceptable (> 5) by the panelists. The panelists felt the difference due to fiber addition, which is in agreement with the results of studies that incorporate vegetable fibers in meat products [46].

## 4. Conclusions

This work demonstrated that CPHF up to 3.0% resulted in being a good substitute for starch for reformulating frankfurters, showing good emulsion stability. In particular, CPHF significantly affects the rheological and composition of the cooked frankfurters with the increase of the total dietary fiber content. TPA analyses showed CPHF fiber contributed to increasing hardness and adhesiveness and decreased cohesiveness in overall samples added with 3% CPHF. Panelists detected clear differences concerning the control frankfurters sausages during the sensory evaluation of the products. Although all the products were considered acceptable, the descriptor texture and flavor were determinant for the panelist to attribute the best score to the samples added with 1.5% CPHF.

## Figures and Tables

**Table 1:** Treatments in the frankfurter elaboration.

Treatment	CPHF (%)	PS (%)
Control	0	3.0
CPHF 1.5 *	1.5	0
CPHF 3.0	3.0	0
CPHF-PS 1.5	1.5	1.5

CPHF: Cacao pod husk flour. PS: Potato starch. * In this treatment, no other ingredient was added to complete the 3.0% generalized in the other treatments.

**Table 2:** Proximate composition (g/100 g sample), TBA value, residual nitrite level, pH, and a_w_ of cacao pod husk and frankfurters.

	CPHF	Frankfurters				
		Control	CPHF 1.5	CPHF 3.0	CPHF-PS 1.5	Sig.
Protein (g/100 g)	4.85 ± 0.01	12.82 ± 0.39 a	13.76 ± 0.31 ab	14.32 ± 0.61 b	13.89 ± 0.19 b	*
Lipid (g/100 g)	0.77 ± 0.15	24.43 ± 0.11 c	20.72 ± 0.46 b	16.09 ± 0.10 a	20.01 ± 1.35 b	**
Total dietary fiber (g/100 g)	37.4 ± 1.0	0.06 ± 0.17 a	0.49 ± 0.08 b	0.96 ± 0.19 c	0.52 ± 0.09 b	*
Carbohydrates (g/100 g)	49.7 ± 1.0	6.4 ± 0.4 c	4.8 ± 0.4 b	5.8 ± 0.6 c	3.1 ± 1.3 a	*
Moisture (g/100 g)	1.08 ± 0.20	53.29 ± 0.41 a	57.43 ± 0.06 b	60.02 ± 0.21 c	59.84 ± 0.21 c	**
Ash (g/100 g)	7.3 ± 0.1	2.91 ± 0.03 b	2.75 ± 0.18 ab	2.80 ± 0.01 b	2.57 ± 0.09 a	*
pH	5.47 ± 0.03	6.30 ± 0.03 b	6.36 ± 0.04 b	6.38 ± 0.06 b	6.09 ± 0.06 a	**
a_w_	0.317 ± 0.015	0.941 ± 0.020	0.946 ± 0.010	0.949 ± 0.008	0.954 ± 0.009	n.s.
Residual nitrite level (mg NaNO_2_)/kg		47.51 ± 1.82 b	41.18 ± 1.21 a	47.70 ± 0.23 b	36.46 ± 4.62 a	*
TBA * (mg MDA/kg product)		0.18 ± 0.03 a	0.25 ± 0.04 b	0.37 ± 0.09 c	0.28 ± 0.07 bc	*

* TBA, thiobarbituric acid value; MDA, malonaldehyde. Results are expressed as means of three samples ± standard deviations. Different letters in the same row indicate significant differences (LSD test, *p* < 0.05). Asterisks indicate significance at * *p* < 0.05; ** *p* < 0.01; n.s. not significant. CPHF Dp < 200 µm.

**Table 3:** Color parameters of cocoa pods husk and frankfurters.

	L*	a*	b*	C*	*h_ab_*	ΔE*
Cocoa pod husk						
Dry	78.92 ± 1.24	4.02 ± 0.35	25.23 ± 0.46	25.55 ± 0.50	80.96 ± 0.59	
Hydrated	53.25 ± 0.68	4.56 ± 0.69	22.95 ± 1.63	23.40 ± 1.74	78.81 ± 0.87	
Frankfurters						
Control	69.32 ± 0.69	6.42 ± 0.15 ab	10.19 ± 0.16 a	12.05 ± 0.17 a	57.79 ± 0.58 a	-
CPHF 1.5	70.63 ± 0.83	6.23 ± 0.37 ab	11.25 ± 0.18 b	12.87 ± 0.25 b	61.05 ± 1.45 b	1.82 ± 0.59 ab
CPHF 3.0	69.25 ± 0.86	6.53 ± 0.19 b	12.31 ± 0.41 c	13.94 ± 0.39 c	62.05 ± 0.99 b	2.28 ± 0.35 a
CPHF-PS 1.5	69.74 ± 0.14	6.04 ± 0.08 a	11.46 ± 0.15 b	12.96 ± 0.17 b	62.22 ± 0.08 b	1.40 ± 0.08 b
Significance	n.s.	*	*	*	**	*

L*, lightness; a*, red/green coordinate; b*, yellow/blue coordinate; C*, chroma; H*, hue; color differences ΔE*. Results are expressed as means of four samples ± standard deviations. Different letters in the same column (only frankfurters) indicate significant differences (LSD test, *p* < 0.05). Asterisks indicate significance at * *p* < 0.05; ** *p* < 0.01; n.s. not significant. CPHF Dp < 200 µm.

**Table 4:** Reflectance ratios R630/R580, R650/R570, and R560/R500 in frankfurter samples.

Treatments	R630/R580	R650/R570	R560/R500
Control	1.53 ± 0.01 b	1.64 ± 0.02 b	0.94 ± 0.00 b
CPHF 1.5	1.47 ± 0.03 a	1.58 ± 0.04 a	0.98 ± 0.01 a
CPHF 3.0	1.48 ± 0.02 a	1.61 ± 0.02 a	1.00 ± 0.00 a
CPHF-PS 1.5	1.47 ± 0.01 a	1.58 ± 0.01 a	0.98 ± 0.00 a
Significance	*	*	*

Results are expressed as means ± standard deviations. Different letters in the same column indicate significant differences (LSD test, *p* < 0.05). Asterisks indicate significance at * *p* < 0.05; n.s. not significant. CPHF Dp < 200 µm.

**Table 5:** Texture profile analysis (TPA) parameters of frankfurters.

Parameter	Control	CPHF 1.5	CPHF 3.0	CPHF-PS 1.5	Sign.
Hardness (N)	81.82 ± 8.29 a	94.63 ± 15.07 b	86.30 ± 8.15 ab	85.60 ± 8.16 ab	**
Adhesiveness	0.20 ± 0.04 a	1.07 ± 0.12 c	0.72 ± 0.24 b	0.62 ± 0.15 b	*
Springiness (mm)	0.29 ± 0.02 b	0.24 ± 0.02 a	0.27 ± 0.01 b	0.28 ± 0.02 b	*
Cohesiveness	0.76 ± 0.01 b	0.75 ± 0.04 b	0.69 ± 0.11 a	0.79 ± 0.04 b	*
Gumminess (N)	61.83 ± 6.09 ab	70.90 ± 8.07 b	58.90 ± 4.94 a	65.60 ± 3.54 b	*
Chewiness (N mm)	17.86 ± 2.11	17.05 ± 2.90	15.98 ± 1.60	17.58 ± 1.81	n.s.
Resilience	0.42 ± 0.01	0.43 ± 0.03	0.37 ± 0.07	0.39 ± 0.08	n.s.

*Legend*: Results are expressed as means ± standard deviations. Different letters in the same row indicate significant differences (LSD test, *p* < 0.05). Asterisks indicate significance at * *p* < 0.05; ** *p* < 0.01; n.s. not significant. CPHF Dp < 200 µm.

## Data Availability

The data presented in this study are available on request from the corresponding author.

## Supplementary Materials

The following are available online at https://www.mdpi.com/article/10.3390/foods10061243/s1, Table S1: Proximate composition (g/100 g dry sample) of frankfurters.

## References

[1] Fernández-López J., Viuda-Martos M., Pérez-Alvarez J.A. (2021). Quinoa and chia products as ingredients for healthier processed meat products: Technological strategies for their application and effects on the final product. Curr. Opin. Food Sci..

[2] Fernández-López J., Viuda-Martos M., Sayas-Barberá M.E., de Vera Navarro-Rodríguez C., Lucas-González R., Roldán-Verdú A., Botella-Martínez C., Pérez-Alvarez J.A. (2020). Chia, Quinoa, and Their Coproducts as Potential Antioxidants for the Meat Industry. Plants.

[3] Delgado-Ospina J., Lucas-González R., Viuda-Martos M., Fernández-López J., Pérez-Álvarez J.Á., Martuscelli M., Chaves-López C. (2021). Bioactive compounds and techno-functional properties of high-fiber co-products of the cacao agro-industrial chain. Heliyon.

[4] Viuda-Martos M., López-Marcos M.C., Fernández-López J., Sendra E., López-Vargas J.H., Pérez-Álvarez J.A. (2010). Role of Fiber in Cardiovascular Diseases: A Review. Compr. Rev. Food Sci. Food Saf..

[5] Pérez-Álvarez J.Á., Botella-Martínez C.M., de Vera Navarro-Rodríguez C., Sayas-Barberá E., Viuda-Martos M., Fernández-López J., Sánchez-Zapata E. (2021). A Preliminary Study on the Incorporation of Quinoa Flour in Organic Pumpkin Creams: Effect on the Physicochemical Properties. Proceedigs.

[6] Lim S.S., Vos T., Flaxman A.D., Danaei G., Shibuya K., Adair-Rohani H., Amann M., Anderson H.R., Andrews K.G., Aryee M. (2012). A comparative risk assessment of burden of disease and injury attributable to 67 risk factors and risk factor clusters in 21 regions, 1990-2010: A systematic analysis for the Global Burden of Disease Study 2010. Lancet.

[7] Föste M., Verheyen C., Jekle M., Becker T. (2020). Fibres of milling and fruit processing by-products in gluten-free bread making: A review of hydration properties, dough formation and quality-improving strategies. Food Chem..

[8] Ribeiro J.S., Santos M.J.M.C., Silva L.K.R., Pereira L.C.L., Santos I.A., da Silva Lannes S.C., da Silva M.V. (2019). Natural antioxidants used in meat products: A brief review. Meat Sci..

[9] Das A.K., Nanda P.K., Madane P., Biswas S., Das A., Zhang W., Lorenzo J.M. (2020). A comprehensive review on antioxidant dietary fibre enriched meat-based functional foods. Trends Food Sci. Technol..

[10] Chaves-López C., Serio A., Mazzarrino G., Martuscelli M., Scarpone E., Paparella A. (2015). Control of household mycoflora in fermented sausages using phenolic fractions from olive mill wastewaters. Int. J. Food Microbiol..

[11] Longato E., Meineri G., Peiretti P.G., Gai F., Viuda-Martos M., Pérez-Álvarez J.Á., Amarowicz R., Fernández-López J. (2019). Effects of hazelnut skin addition on the cooking, antioxidant and sensory properties of chicken burgers. J. Food Sci. Technol..

[12] Mehta N., Ahlawat S.S., Sharma D.P., Dabur R.S. (2015). Novel trends in development of dietary fiber rich meat products—A critical review. J. Food Sci. Technol..

[13] Lu F., Rodriguez-Garcia J., Van Damme I., Westwood N.J., Shaw L., Robinson J.S., Warren G., Chatzifragkou A., McQueen Mason S., Gomez L. (2018). Valorisation strategies for cocoa pod husk and its fractions. Curr. Opin. Green Sustain. Chem..

[14] International Cocoa Organization ICCO (2020). Quarterly Bulletin of Cocoa Statistics.

[15] Campos-Vega R., Nieto-Figueroa K.H., Oomah B.D. (2018). Cocoa (*Theobroma cacao* L.) pod husk: Renewable source of bioactive compounds. Trends Food Sci. Technol..

[16] Yapo B.M., Besson V., Koubala B.B., Koffi K.L. (2013). Adding Value to Cacao Pod Husks as a Potential Antioxidant-Dietary Fiber Source. Am. J. Food Nutr..

[17] Vriesmann L.C., de Mello Castanho Amboni R.D., De Oliveira Petkowicz C.L. (2011). Cacao pod husks (Theobroma cacao L.): Composition and hot-water-soluble pectins. Ind. Crop. Prod..

[18] Yusof F., Khanahmadi S., Amid A., Mahmod S.S. (2016). Cocoa pod husk, a new source of hydrolase enzymes for preparation of cross-linked enzyme aggregate. Springerplus.

[19] Fernández-López J., Lucas-González R., Viuda-Martos M., Sayas-Barberá E., Navarro C., Haros C.M., Pérez-Álvarez J.A. (2019). Chia (Salvia hispanica L.) products as ingredients for reformulating frankfurters: Effects on quality properties and shelf-life. Meat Sci..

[20] Horwitz W. (2000). Official Methods of Analysis of AOAC International.

[21] International Organization for Standardization (1975). Meat and Meat Products: Determination of Nitrite Content.

[22] Rosmini M.R., Perlo F., Pérez-Alvarez J.A., Pagán-Moreno M.J., Gago-Gago A., López-Santoveña F., Aranda-Catalá V. (1996). TBA test by an extractive method applied to “Paté. ” Meat Sci..

[23] Association A.M.S., AMSA (2012). Meat Color Measurement Guidelines.

[24] Sánchez-Zapata E., Fuentes-Zaragoza E., de Vera Navarro-Rodríguez C., Sayas E., Sendra E., Fernández-López J., Pérez-Alvarez J.A. (2011). Effects of tuna pâté thickness and background on CIEL∗a∗b∗ color parameters and reflectance spectra. Food Control.

[25] Muñoz-Almagro N., Valadez-Carmona L., Mendiola J.A., Ibáñez E., Villamiel M. (2019). Structural characterisation of pectin obtained from cacao pod husk. Comparison of conventional and subcritical water extraction. Carbohydr. Polym..

[26] Viuda-Martos M., Fernández-López J., Sayas-Barbera E., Sendra E., Navarro C., Pérez-Álvarez J.A. (2009). Citrus Co-Products as Technological Strategy to Reduce Residual Nitrite Content in Meat Products. J. Food Sci..

[27] Pérez-Rodríguez M.L., Bosch-Bosch N., Garciá-Mata M. (1996). Monitoring nitrite and nitrate residues in frankfurters during processing and storage. Meat Sci..

[28] Merino L., Darnerud P., Toldrá F., Ilbäck N.-G. (2016). Time-dependent depletion of nitrite in pork-beef and chicken meat products affects nitrite intake estimation. Food Addit. Contam. Part A.

[29] Martín León V., Luzardo O.P. (2020). Evaluation of nitrate contents in regulated and non-regulated leafy vegetables of high consumption in the Canary Islands, Spain: Risk assessment. Food Chem. Toxicol..

[30] Lebrun S., Van Nieuwenhuysen T., Crèvecoeur S., Vanleyssem R., Thimister J., Denayer S., Jeuge S., Daube G., Clinquart A., Fremaux B. (2020). Influence of reduced levels or suppression of sodium nitrite on the outgrowth and toxinogenesis of psychrotrophic Clostridium botulinum Group II type B in cooked ham. Int. J. Food Microbiol..

[31] Garrote G., Cruz J.M., Moure A., Domínguez H., Parajó J.C. (2004). Antioxidant activity of byproducts from the hydrolytic processing of selected lignocellulosic materials. Trends Food Sci. Technol..

[32] Sheard P.R., Enser M., Wood J.D., Nute G.R., Gill B.P., Richardson R.I. (2000). Shelf life and quality of pork and pork products with raised n-3 PUFA. Meat Sci..

[33] Falowo A.B., Fayemi P.O., Muchenje V. (2014). Natural antioxidants against lipid–protein oxidative deterioration in meat and meat products: A review. Food Res. Int..

[34] Hernández B., Sáenz C., Alberdi C., Diñeiro J.M. (2016). CIELAB color coordinates versus relative proportions of myoglobin redox forms in the description of fresh meat appearance. J. Food Sci. Technol..

[35] Shimokomaki M., Youssef Youssef E., Terra N., Caballero B., Finglas P., Toldra F. (2003). Curing. Encyclopedia of Food Sciences and Nutrition.

[36] Hernández Salueña B., Sáenz Gamasa C., Diñeiro Rubial J.M., Alberdi Odriozola C. (2019). CIELAB color paths during meat shelf life. Meat Sci..

[37] Martín-Sánchez A.M., Sánchez-Zapata E., Viuda-Martos M., Sendra E., Sayas-Barberá E., Fernández-López J., Pérez-Álvarez J.Á., Chaves-López C. (2010). Influencia de la adición de sorbato sobre los cocientes de reflectancia R650/R570, R560/R500 y R630/R580 en productos cárnicos crudo-curados. Opt. Pura y Apl..

[38] Strange E.D., Benedict R.C., Gugger R.E., Metzger V.G., Swift C.E. (1974). Simplified Methodology for Measuring Meat Color. J. Food Sci..

[39] Sánchez-Zapata E., Zunino V., Pérez-Alvarez J.A., Fernández-López J. (2013). Effect of tiger nut fibre addition on the quality and safety of a dry-cured pork sausage (“Chorizo”) during the dry-curing process. Meat Sci..

[40] Cruz A.G., Cadena R.S., Walter E.H.M., Mortazavian A.M., Granato D., Faria J.A.F., Bolini H.M.A. (2010). Sensory analysis: Relevance for prebiotic, probiotic, and synbiotic product development. Compr. Rev. Food Sci. Food Saf..

[41] Biswas A.K., Kumar V., Bhosle S., Sahoo J., Chatli M.K. (2011). Dietary fibers as functional ingredients in meat products and their role in human health. Int. J. Livest. Prod..

[42] Ahmad S.S., Khalid M., Younis K. (2020). Interaction study of dietary fibers (pectin and cellulose) with meat proteins using bioinformatics analysis: An In-Silico study. LWT.

[43] Martínez J.A., Melgosa M., Pérez M.M., Hita E., Negueruela A.I. (2001). Note. Visual and Instrumental Color Evaluation in Red Wines. Food Sci. Technol. Int..

[44] Cook S.L., Woods S., Methven L., Parker J.K., Khutoryanskiy V. (2018). Mucoadhesive polysaccharides modulate sodium retention, release and taste perception. Food Chem..

[45] Sun C., Zhou X., Hu Z., Lu W., Zhao Y., Fang Y. (2020). Food and salt structure design for salt reducing. Innov. Food Sci. Emerg. Technol..

[46] Pintado T., Herrero A.M., Jiménez-Colmenero F., Ruiz-Capillas C. (2016). Strategies for incorporation of chia (Salvia hispanica L.) in frankfurters as a health-promoting ingredient. Meat Sci..

