# Supplementary material for: Cacao Pod Husk Flour as an Ingredient for Reformulating Frankfurters: Effects on Quality Properties
Source: Foods. 2021 May 30;10(6):1243. doi: 10.3390/foods10061243 (PMC8229612; doi:10.3390/foods10061243)
Supplement: Supplementary file 1 [file foods-10-01243-s001.zip › foods-1213457-supplementary.pdf]

Table S1. Proximate composition (g/100 g dry sample) of frankfurters.

	Frankfurters				
	Control	CPHF 1.5	CPHF 3.0	CPHF-PS 1.5	Sig.
Protein (g/100 g)	27.45 ± 0.83 a	32.32 ± 0.66 ab	35.82 ± 1.31 b	34.59 ± 0.41 b	*
Lipid (g/100 g)	52.30 ± 0.24 c	48.67 ± 0.98 b	40.25 ± 0.21 a	49.83 ± 2.89 b	**
Total dietary Fiber (g/100 g)	0.13 ± 0.36 a	1.15 ± 0.17 b	2.40 ± 0.41 c	1.29 ± 0.19 b	*
Carbohydrates (g/100 g)	13.7 ± 0.9 c	11.28 ± 0.86 b	14.5 ± 1.3 c	7.72 ± 2.8 a	*
Ash (g/100 g)	6.23 ± 0.06 b	6.46 ± 0.39 ab	7.00 ± 0.02 b	6.40 ± 0.19 a	*

Results are expressed as means of three samples ± standard deviations. Different letters in the same row indicate significant differences (LSD test, $p < 0.05$). Asterisks indicate significance at *$p < 0.05$; **$p < 0.01$; n.s. not significant. CPHF Dp < 200 μm.
